# Precision Glycan Supplementation Improves Gut Microbiota Diversity, Performance, and Disease Outbreak Resistance in Broiler Chickens

**DOI:** 10.3390/ani14010032

**Published:** 2023-12-21

**Authors:** Edina Lobo, Yadav S. Bajagai, Advait Kayal, Santiago Ramirez, Anja Nikolić, Rolando Valientes, Dragana Stanley

**Affiliations:** 1 Institute for Future Farming Systems, Central Queensland University, Rockhampton, QLD 4702, Australia; edina.lobo@cqumail.com (E.L.); advait.kayal@cqumail.com (A.K.); 2 DSM Firmenich, 4303 Kaiseraugst, Switzerland; santiago.ramirez@dsm.com (S.R.);; 3 Faculty of Veterinary Medicine, University of Belgrade, Bulevar Oslobodjenja 18, 11000 Belgrade, Serbia; anja.nikolic@online.vet.bg.ac.rs

**Keywords:** glycan, poultry, intestinal, precision glycan

## Abstract

**Simple Summary:**

The intensive poultry production system faces production challenges like pathogenic infections, poor performance, and bird welfare. The use of antibiotics has been reduced due to regulations and increasing antimicrobial resistance, promoting research into viable alternatives. Precision glycans represent an alternative that modulates the gut microbial community and its metabolic functions. This study compares birds fed precision glycan-supplemented and non-supplemented diets in a commercial broiler farm. We report major alterations in microbiota across caecum, ileum, and ileum mucosa gut sections. The treated birds also showed better intestinal morphology and higher weight gain with an improvement in feed efficiency and disease resistance.

**Abstract:**

The poultry industry contributes significantly to the global meat industry but faces many production challenges like high-density housing, welfare issues, and pathogenic infections. While antibiotics have commonly been used to treat many of these issues, they are being removed from poultry production globally due to increased microbial resistance. Precision glycans offer a viable alternative to antibiotics by modulating microbial metabolic pathways. In this study, we investigated the effects of precision glycan supplementation on productivity and gut microbiota in broilers. The experiment was conducted in a commercial setting using 32,400 male Ross chickens randomly divided into three sheds with 10,800 birds each. One shed with 12 pen replicates of 900 birds was used as control, while the other two with an equal number of replicates and birds were assigned to precision glycan supplementation. The treatment significantly improved the average daily weight gain and feed conversion ratio, with a significant modification in the abundance of several bacterial taxa in the caecum, ileum, and ileum mucosa microbial communities. There was increased richness and diversity in the caecum, with a reduction in *Proteobacteria* and an increase in *Firmicutes*. Richness remained unchanged in the ileum, with an increase in diversity and reduction in pathogenic genera like *Clostridium* and *Escherichia-Shigella*. Ileum mucosa showed a lower abundance of mucin degraders and an increased presence of next-generation probiotics. Supplemented birds showed a high level of disease resistance when the farm experienced an outbreak of infectious bronchitis, evidenced by lower mortality. Histological analysis confirmed improvements in the ileum and liver health, where the precision glycan supplementation reduced the area of congested sinusoids compared to the control group in the liver and significantly improved ileum intestinal morphology by increasing crypt depth and surface area. These results collectively suggest that precision glycans offer substantial benefits in poultry production by improving productivity, gut health, and disease resistance.

## 1. Introduction

The poultry industry contributes significantly to global meat production as an affordable, high-quality protein source [1]. Chicken meat is a rich and cheap source of nutrients compared to other meat products. The low cost, efficient growth, and short turnover time in chicken production make it an attractive source of protein [1,2]. In 2022, the United States produced 21 million metric tons of broiler meat, followed by Brazil with an estimated 14.5 million metric tons [3]. In Australia, the poultry industry contributed AUD 3.149 billion to the national economy in 2022 [4]. Although the global poultry industry is growing, it faces many production challenges. These include high-density housing facilities, rapid growth rates, and welfare issues. Production challenges result in stressed animals being more susceptible to pathogenic infections. Another contributing factor to disease spread within flocks is the free-range system. Access to the range exposes the flock to environmental pathogens from soil and wild animals, including rodents and birds [5,6]. In many livestock systems, using antibiotics for treating infections, reducing mortality, and stimulating growth was common practice [7,8]. However, antibiotics are no longer a viable option due to widespread antimicrobial resistance [9].

Research into antibiotic alternatives is constantly evolving. Precision biotics (PBs) are a relatively new additive to poultry nutrition. Precision biotics are a specialised category of supplements that incorporate precision-designed biological molecules, including precision glycans, for targeted and specific effects on the host microbiome and overall health. These precision-designed molecules are carefully engineered to interact with specific receptors or functional pathways in the host and/or host microbiome to promote desired physiological responses and modulate both the composition and function of the microbial communities of the supplemented host [10].

The concept of precision biotics expands beyond traditional multi-species probiotics by incorporating precise, predesigned, and customised biological molecules that include glycans, peptides, proteins, or other biologically active compounds. Recent studies have indicated the possible use of glycan-based precision biotics in modulating metabolic pathways in humans as well as animals [11,12,13].

Glycans are carbohydrate polymers formed by the linkage of monosaccharides. They also form important biomolecules with lipids and proteins [14]. The biological roles of glycans can be summarised into three main areas: (1) structural like cellulose that comprises cell walls in plants, (2) energy metabolism as a carbohydrate reserve, and (3) as information transmitters like molecular patterns recognised by glycan-binding proteins (GBPs). In eukaryotic cells, glycans protect and stabilise cells by forming barriers. Proteoglycans are key molecules in multicellular organisms for maintaining the structure and integrity of tissues [15]. Glycans also play a vital role in protein folding and maintaining protein physical properties like conformation and solubility [14]. Plant and animal glycan polymers also function to sequester and/or store nutrients [15]. The multi-functional nature of glycans makes them an attractive alternative to standard treatment strategies. The advances in synthetic glycan development suggest that altering the valency of glycan scaffolds could create potential bacterial inhibitors [16,17]. The surface of epithelial cells is covered by a gel-like layer composed of glycoconjugates, which exhibit various carbohydrate epitopes. These epitopes mediate a variety of cell functions via carbohydrate–lectin interactions. Some viral and bacterial pathogens bind to carbohydrate motifs present on epithelial cell surfaces via lectins and thus cause infection in humans [18]. By understanding the molecular mechanisms behind the glycan–lectin interaction, synthetic glycans that can mimic epithelial cell surfaces can be designed to inhibit pathogen attachment [18,19].

The glycans formed via partial synthesis due to abnormal glycosylation in cancer cells can be used in cancer therapies like glycan-based vaccines [20,21]. These vaccines could potentially mount an immune response against the altered glycan structures [22]. Several glycan-based vaccines have been undergoing clinical trials with promising outcomes [23,24,25,26].

Mucin is a major component of the epithelial cell layer that is primarily composed of O-glycans linked to amino acids serine or threonine [27]. In the intestine, the mucus layer serves as a protective barrier, which is divided into an outer layer in direct contact with microbes and an inner layer adjacent to epithelia [28,29]. This feature of the mucus layer facilitates host–microbe interactions while preventing direct bacterial adhesion and severe infections [30,31,32]. Along with this protective feature of glycans, they can also influence the gut microbiota composition by serving as fermentation substrates, producing short-chain fatty acids, further influencing health, immunity, and disease resistance. Glycan preferences differ based on bacterial species; hence, it is possible to influence the presence and proliferation of desired microbial groups by supplementing specific glycans [11]. Research reporting a range of benefits from using natural glycan-based prebiotics provides confidence that precision-designed glycans will also offer multiple health and metabolic benefits to the host.

In addition to well-researched benefits and various applications of precision glycans in human health, our understanding of an equally extensive range of precision glycan health benefits on the health and nutrition of broiler chickens is also accumulating. A study conducted on broilers over multiple trials with two structurally distinct precision glycan indicated that different glycan structures altered different aspects of bird performance by positively modulating metabolic pathways of the gut microbiome. This study suggests that glycan-based metabolic modulators could target pathways that benefit broiler productivity, sustainability, and welfare [33]. Another research group indicated that supplementing feed with a glycan-based precision biotic improved broiler performance and reduced footpad lesions due to reduced ammonia and pH, and improved litter quality [13]. A recent study also evaluated the effect of precision glycan supplements on bird performance under enteric stress. They observed that bird growth and intestinal health markedly improved, suggesting that broilers provided with supplemented feed were more resistant to enteric stress [12].

This study aimed to use a proprietary precision glycan to investigate its effect on pathogen load and the development of intestinal microbial communities in broilers. Our data indicates that the precision glycan supplementation improved bird performance, mortality, intestinal health, and disease resistance.

## 2. Materials and Methods

### 2.1. Animal Trial

The study was conducted using 32,400 male Ross 308 broilers. All birds received in ovo vaccines against Newcastle disease (ND) and Infectious Bursal Disease (IBD) and were vaccinated against Infectious Bronchitis (IB), ND, and IBQX at day old. All birds were also vaccinated against ND+IBD on day ten and against IB on day 16. The feed and water were available ad libitum. The houses were controlled by an evaporative cooling system with tunnel ventilation, fed by an automatic feeder and a drip irrigation system. 

The chicks were randomly assigned to two treatment groups. One house with 10,800 birds was allotted to control (CT), and two houses, each with 10,800 birds, were assigned to treatment (PB.A and PB.B). Each house had 12 replicates, with 900 birds randomly assigned to each replicate. The feed supplied was according to breed recommendation. Feed did not contain any in-feed antibiotic growth-promoting substance or ionophores. The diet of the treatment group was supplemented with a precision biotic (PB) (Symphiome™, DSM Firmenich Animal Nutrition and Health, Kaiseraugst, Switzerland) at the rate of 900 g per ton of feed for treatment groups. Phase feeding was followed with Starter from 0 to 10 days, Grower from 11 to 22 days, Finisher from 23 to 35 days, and Withdrawal from 36 to 38 days. The performance parameters were measured after each phase of the feeding. For the performance monitoring during the trial, average bird weight was based on a random sample of 100 birds per replicate pen, representing more than 10% of birds in the shed. The experiment lasted for 38 days. The final performance data for 0–38 days, shown in Table 1, are based on the final bird weights of the entire flock collected automatically at the processing facility. FCR was calculated by dividing feed intake by the body weight.

### 2.2. DNA Extraction and Amplicon Sequencing

At day 38, 72 birds (6 birds per replicate) were selected at random from each house and samples were collected from caecum, ileum, and ileum mucosa. The DNA was extracted using a DNA mini spin column (Enzymax LLC, CAT#EZC101, Lexington, KY, USA). The concentration and quality of extracted DNA were measured using a NanoDrop One UV-Vis spectrophotometer (ThermoFisher Scientific, Waltham, MA, USA). The following primers specific to the V3-V4 region of the 16S rRNA gene were used with spacers, barcodes, and Illumina sequencing linkers [34]. Pro341F (5′-CCTACGGGNBGCASCAG-3′) was the forward primer, and 805R (5′-GACTACNVGGGTATCTAATCC-3′) was the reverse. The 16S amplicon library was then purified using AMPure XP kits (Beckman Coulter, Brea, CA, USA) and sequenced using Illumina MiSeq platform 2 × 250 bp paired-ended configuration.

### 2.3. Data Analysis and Bioinformatics

A total of 199 samples were successfully sequenced. The better-quality reads were further processed using a minimum Phred score of 20 across a 200 nt length. Cutadapt was used for demultiplexing the raw DNA sequences [35], and Quantitative Insights into Microbial Ecology 2 (QIIME 2) was used for analysis [36]. The filtering, denoising, and chimaera removal were conducted using DADA2 with default parameters [37]. Taxonomy was assigned using the SILVA v 138.1 database as a reference [38,39]. OTU clusters were formed from the ASV data at 98% similarity. The data were rarefied at a minimum of 3000 sequences per sample for complete analysis and interpretation. R packages, including Phylosmith (https://schuyler-smith.github.io/phylosmith/, accessed on 12 December 2023), Phyloseq (https://joey711.github.io/phyloseq/, accessed on 12 December 2023), and Microeco (https://chiliubio.github.io/microeco/, accessed on 12 December 2023), were used for downstream analysis and visualisation of the data.

### 2.4. Histology

The samples for histology were collected from the ileum and fixed in 10% neutral buffered formalin. The tissue processing involved fixation, embedding in paraffin, and cutting with microtome. The staining was performed using Hematoxylin and Eosin staining (H&E). Twenty samples were collected across control and treatment groups for histology.

## 3. Results

### 3.1. Animal Health and Performance

The control flock showed symptoms of infectious bronchitis on day 19, while the onset in the precision glycan treatment flocks was delayed by ten days. Consequently, the mortality at day 38 in the control flock was significantly higher at 30.2% compared to only 12.75% for the treatment group. Overall, the precision biotic-treated birds recorded an average daily weight gain of 70.0 g, which is significantly higher (*p* < 0.001) than the average gain in the control group (67.86 g). The FCR of the precision biotic-treated group was significantly improved by an average of 11 points (1.69 in the control vs. 1.58 in the PB group, *p* < 0.0001). In addition, total feed intake per bird and average daily feed intake were significantly lower in the PB group compared to the control. However, during the period 0–10 days, the performance by FCR was better in the control group compared to either of the treatment sheds.

There were no differences in the footpad lesion score and carcass traits except the significantly higher proportion (3.76% in control vs. 3.46% in PB, *p* < 0.0001) of gizzard giblets in the control group compared to the PB group. The performance parameters of birds are presented in Table 1.

### 3.2. Overall Microbial Community Structure

**Figure 1:**
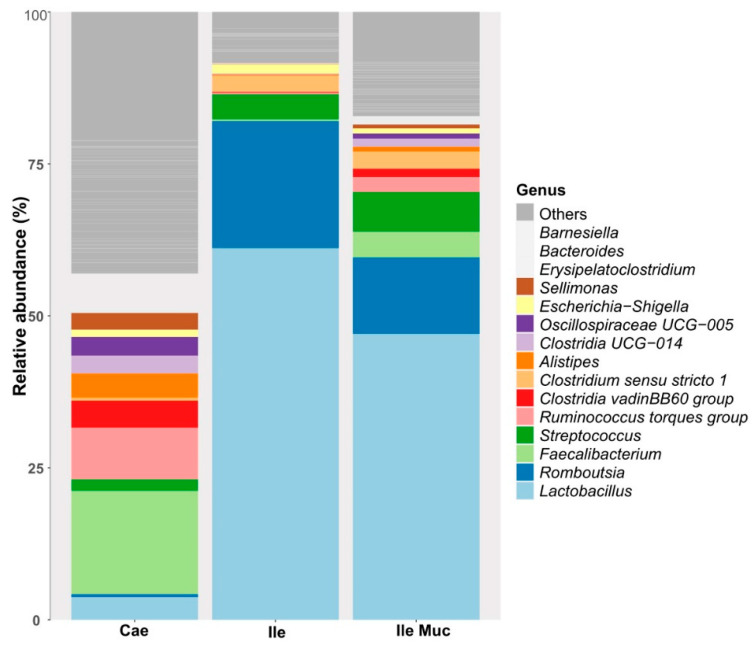
The most abundant genera of the intestinal gut sections of the experimental birds. The plot shows the relative abundance of the 15 most abundant genera. Cae = Caecum, Ile = Ileum, and Ile Muc = Ileum mucosa.

The microbial communities present in the samples collected from the caecum, ileum, and ileum mucosa were mainly assigned to phylum *Firmicutes*, followed by *Bacteroidota* in the caecum and *Proteobacteria* and *Actinobacteria* in all three sections. The lower abundant phylum included *Cyanobacteria*, *Desulfobacterota*, *Chloroflexi*, and *Verrucomicrobiota*. The abundant genera within all sections were *Lactobacillus*, *Romboutsia*, *Streptococcus*, *Faecalibacterium*, *Escherichia-Shigella*, *Bacteroides*, *Alistipes*, *Sellimonas*, *Barnesiella*, *Erysipelatoclostridium*, *Oscilliospiraceae UCG-005*, *Clostridia UCG-014*, *Clostridium sensu stricto 1*, *Clostridium vadinBB60 group*, and *Ruminococcus torques group* (Figure 1).

### 3.3. Caecal Microbiota

**Figure 2:**
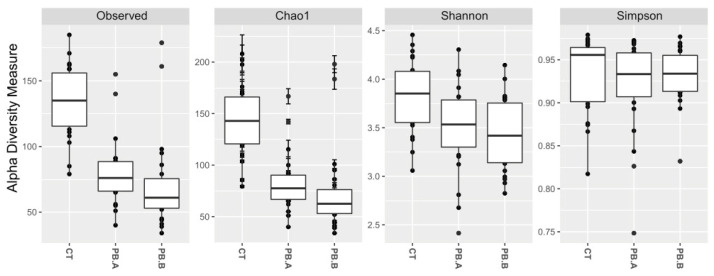
Alpha diversity of caecal microbiota. CT indicates an unsupplemented control shed; PB.A and PB.B are the two sheds where the animals were supplemented with 900 g/t of PB (precision biotic) for the duration of the trial. Observed: CT vs. PB (*p* < 0.0001), Observed: CT vs. PB.A (*p* < 0.0001), Observed: CT vs. PB.B (*p* < 0.0001), Chao1: CT vs. PB (*p* < 0.0001), Chao1: CT vs. PB.A (*p* < 0.0001), Chao1: CT vs. PB.B (*p* < 0.0001), Shannon: CT vs. PB (*p* < 0.005), Shannon: CT vs. PB.A (*p* < 0.05), Shannon: CT vs. PB.B (*p* < 0.005), Simpson: CT vs. PB (*p* > 0.05), Simpson: CT vs. PB.A (*p* > 0.05), Simpson: CT vs. PB.B (*p* > 0.05).

The major caecal microbiota consisted of the following genera *Barnesiella*, *Bacteroides*, *Erysipelatoclostridium*, *Sellimonas*, *Escherichia*-Shigella, *Oscilliospiraceae UCG-005*, *Clostridia UCG-014*, *Alistipes*, *Clostridium sensu stricto 1*, *Clostridia vadinBB60 group*, *Ruminococcus torques group*, *Streptococcus*, *Faecalibacterium*, *Romboutsia*, and *Lactobacillus*. The alpha and beta diversity were examined to compare the microbial communities between treatment and control groups. The precision biotic treatment group showed a reduction in richness (*p* < 0.0001) and diversity (*p* < 0.005) compared to the caecum of the control group (Figure 2).

**Figure 3:**
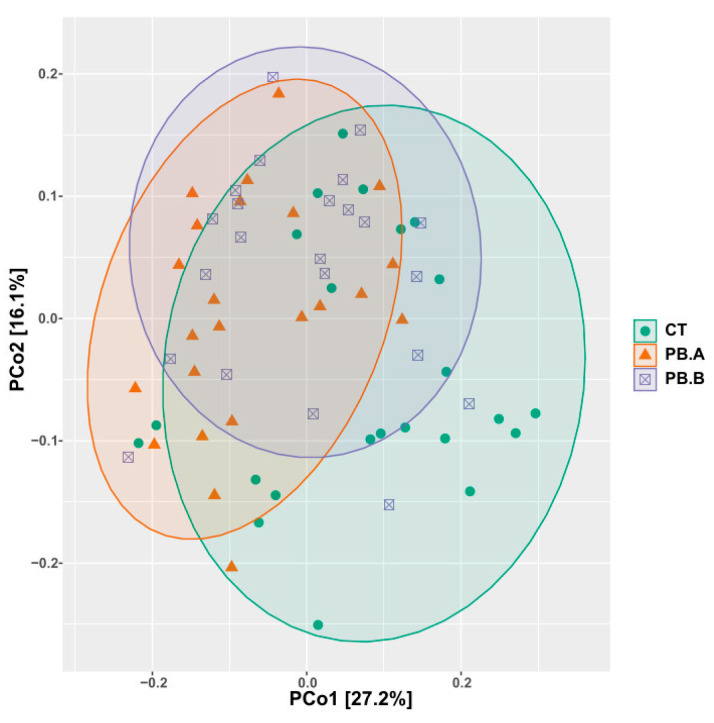
Sample-to-sample and group relationships in caecum are presented by a Weighted UniFrac distance-based PCoA plot. CT = Control; PB.A = Precision Biotic shed A; PB.B = Precision Biotic shed B.

PERMANOVA multivariate analysis based on Weighted (WUF) and Unweighted UniFrac (UW UF) distances showed that caecal microbial communities were significantly different between CT and PB, and paired MANOVA analysis between all groups, including all three sheds, confirmed differences between CT and each of the two PB sheds (both *p* < 0.001). However, differences also existed between the two PB-supplemented sheds PB.A vs. PB.B (WUF *p* = 0.002, UW UF *p* < 0.001) as visualised in Figure 3.

**Figure 4:**
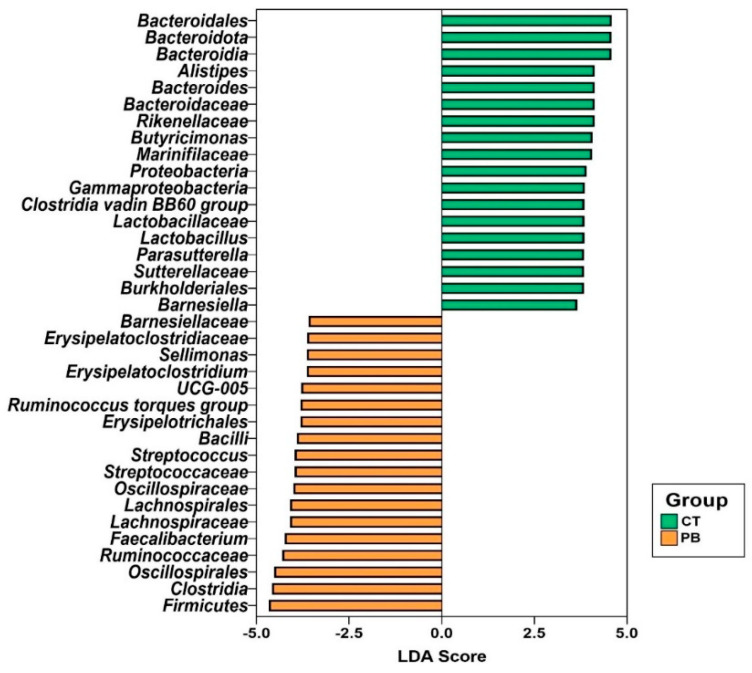
LEfSe plot of differential taxa in caecum. The plot shows all significant taxonomic levels. CT = Control; PB = Precision Biotic.

The linear discriminant analysis (LDA) effect size (LEfSe) tool was used to determine statistically and biologically relevant microbial biomarkers for each group (Figure 4). *Proteobacteria* and *Firmicutes* are phylum-level markers for control and treatment groups, respectively. In the caecum, precision biotic increased the relative abundance of *Firmicutes* while reducing the abundance of *Proteobacteria*.

### 3.4. Ileum Microbiota

**Figure 5:**
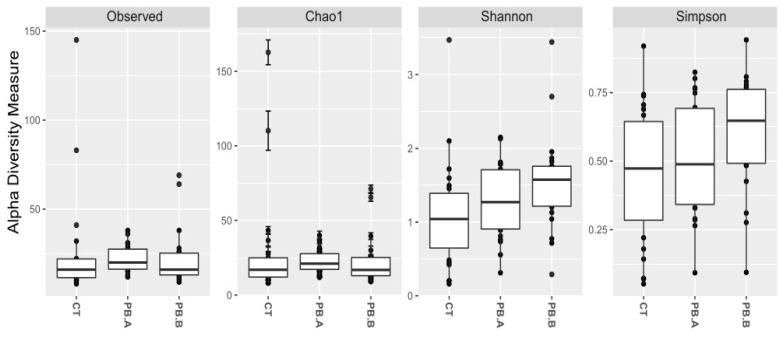
Alpha diversity of ileum content microbiota. CT indicates an unsupplemented control shed; PB.A and PB.B are the two sheds where the animals were supplemented with 900 g/t of PB (precision glycan) for the duration of the trial. Observed: CT vs. PB (*p* > 0.05), Observed: CT vs. PB.A (*p* > 0.05), Observed: CT vs. PB.B (*p* > 0.05), Chao1: CT vs. PB (*p* > 0.05), Chao1: CT vs. PB.A (*p* > 0.05), Chao1: CT vs. PB.B (*p* > 0.05), Shannon: CT vs. PB (*p* < 0.05), Shannon: CT vs. PB.A (*p* > 0.05), Shannon: CT vs. PB.B (*p* < 0.01), Simpson: CT vs. PB (*p* > 0.05), Simpson: CT vs. PB.A (*p* > 0.05), Simpson: CT vs. PB.B (*p* < 0.05).

In order of relative abundance, the core microbiota of ileum includes the genera *Lactobacillus*, *Romboutsia*, *Streptococcus*, *Corynebacterium*, and *Escherichia-Shigella*. The alpha diversity profile indicates that the precision biotic does not affect richness but increases the diversity of the ileum microbial community (Figure 5).

**Figure 6:**
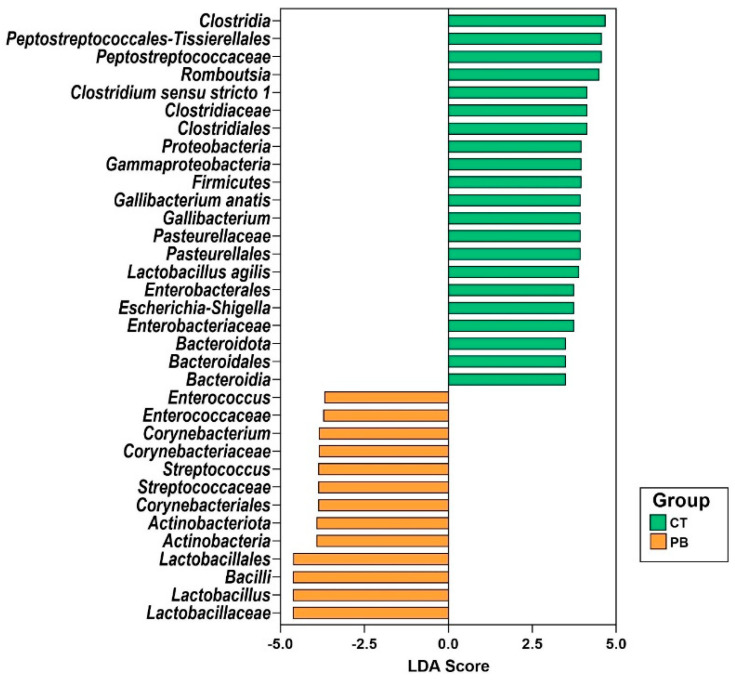
LEfSe plot of differential taxa in ileum. The plot shows all taxonomic levels. CT = Control, PB = Precision Biotic.

As observed in the caecum, in ileum microbiota, multivariate analysis (PERMANOVA) showed significant differences (*p* < 0.05) between CT and PB when compared with unweighted unifrac distances. However, there were no differences (*p* > 0.05) in weighted unifrac distances. LefSe analysis of Ileum microbiota showed that precision biotic reduced *Proteobacteria*, *Escherichia-Shigela*, *Gallibacterium anatis*, and *Clostridium* (Figure 6).

### 3.5. Ileum Mucosa Microbiota

**Figure 7:**
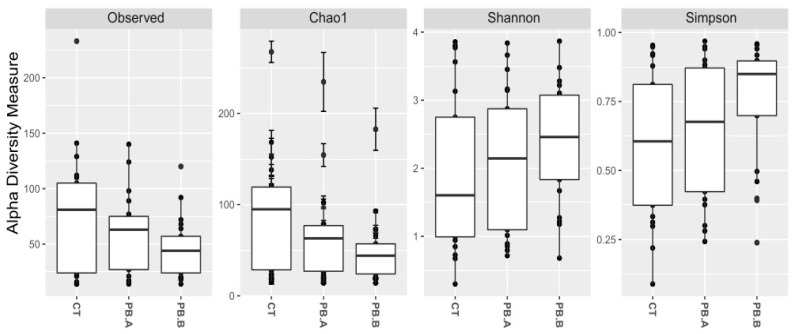
Alpha diversity of ileum mucosa microbial community. CT indicates an unsupplemented control shed; PB.A and PB.B are the two sheds where the animals were supplemented with 900 g/t of PB for the duration of the trial. Observed: CT vs. PB (*p* > 0.05), Observed: CT vs. PB.A (*p* > 0.05), Observed: CT vs. PB.B (*p* > 0.05), Chao1: CT vs. PB (*p* < 0.05), Chao1: CT vs. PB.A (*p* > 0.05), Chao1: CT vs. PB.B (*p* < 0.05), Shannon: CT vs. PB (*p* > 0.05), Shannon: CT vs. PB.A (*p* > 0.05), Shannon: CT vs. PB.B (*p* > 0.05), Simpson: CT vs. PB (*p* > 0.05), Simpson: CT vs. PB.A (*p* > 0.05), Simpson: CT vs. PB.B (*p* > 0.05).

The core microbiota in ileum mucosa consisted of *Lactobacillus*, *Romboutsia*, *Faecalibacterium*, and *Streptococcus.* The alpha diversity in ileum mucosa samples displayed an unusual pattern with a decrease in richness and an increase in diversity (Figure 7).

**Figure 8:**
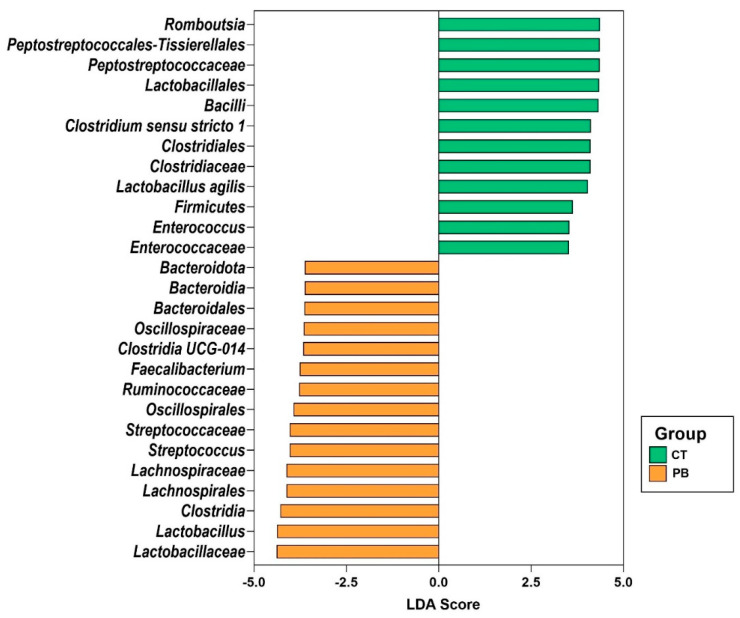
LEfSe plot of genus level differential taxa in ileum mucosa. The plot shows all significant taxonomic levels. CT = Control; PB = Precision Biotic.

There were no significant alterations between the groups or sheds based on either weighted or unweighted UniFrac (PERMANOVA *p* > 0.05). LEfSe analysis indicates an increased presence of *Clostridium* in control compared to treatment in ileum mucosa (Figure 8).

### 3.6. Multivariate Analysis

Multivariate PERMANOVA analyses were presented for each gut section above. Considering the complete dataset, using both weighted and unweighted UniFrac, gut origin and the shed show significant influence on microbial communities, while the PB treatment strongly altered microbial membership by UWU and marginally (*p* = 0.06) affected the membership of microbiota estimated by weighted UniFrac (Table 2).

### 3.7. Intestinal Morphology

**Figure 9:**
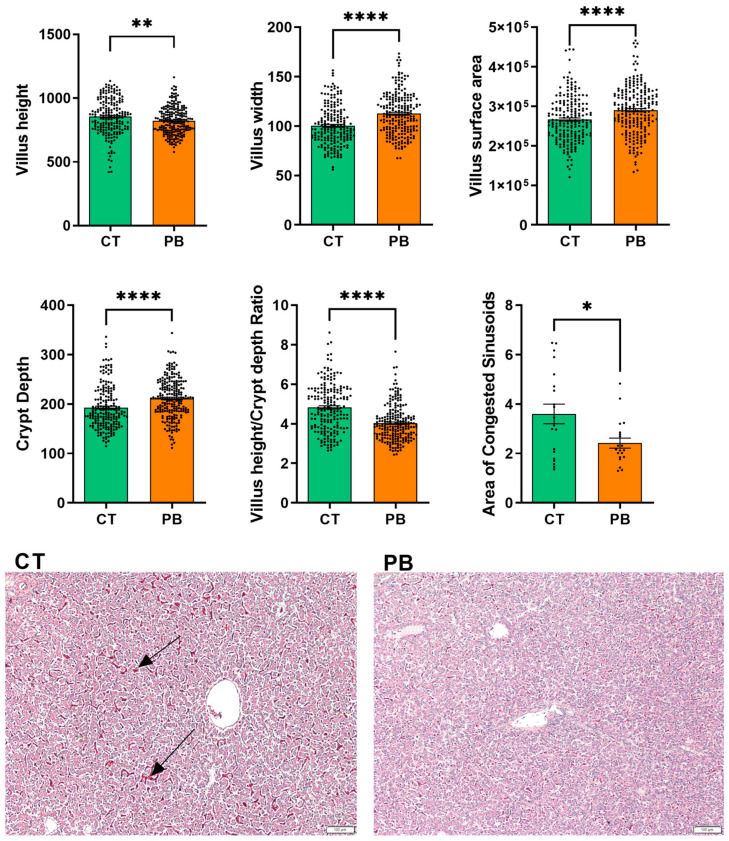
Ileum histology shows significant alterations in the ileum and liver morphology. PB reduced the villus height, increased the villus width and surface area, increased crypt depth, reduced villus height/crypt depth ratio, and reduced the area of congested sinusoids in the liver. Histological images show highly congested sinusoids (indicated by arrows) in CT livers compared to PB. CT = Control; PB = Precision Biotic (precision glycan). * = *p* < 0.05, ** = *p* < 0.01, **** = *p* < 0.0001.

The morphology of ileal mucosa was well preserved in both groups. However, the treated group showed notable alterations in the structural organisation of villi and crypts compared to the control group (Figure 9). Villus height was significantly lower (*p* = 0.002) in the treated group, but the villi were wider (*p* < 0.0001) compared to that in the control. Consequently, the villus surface area was significantly larger in the treated group (*p* < 0.0001). Crypt depth was significantly higher in the treated group (*p* < 0.0001). The presence of higher villi and shallower crypts in the control group led to a significantly higher villus height to crypt depth ratio (*p* < 0.0001). Histological analysis of liver tissue showed no histological appearance of pathological changes; however, the control group had a significantly larger area of congested sinusoids (Figure 9) compared to the treated group (*p* < 0.046).

## 4. Discussion

The manipulation of poultry gut microbiota for improving chicken welfare and production has been an evolving area of research. The gut microbiota plays an essential role in physiological processes like nutrient absorption, immunity, digestion, etc., significantly contributing to overall health [40]. Many alternative supplements like probiotics, prebiotics [41], phytogens [42], and organic acids [43] have shown promising results in improving chicken health when compared to antibiotics [44]. However, altering the microbiota composition of the gut alone may not be sufficient. The precise control of microbial metabolic pathways involved in improved performance and pathogenicity is the next challenge for optimal gut function. Precision glycans can modulate the gut microbiome to produce beneficial metabolites and influence nutrient metabolism [33].

The precision biotics used in this study affected the alpha diversity. In caecum, we observe perturbations towards reduced richness and diversity in PB groups. In the ileum, the precision biotic increased diversity with no notable effect on richness, meaning that the number of different ileal species did not change significantly, but their proportions and interactions were altered. In the ileum mucosa, Observed and Chao1 indices implied a reduction in species richness, while Shannon and Simpson indices showed an increase in species diversity. This suggests a reduction in the total number of species but an increase in their sphere of influence in the ileum mucosa. The PB effect on alpha diversity could be described as potentially gut health-promoting. Unlike the increase in diversity, generally regarded as beneficial, the sudden rise of species richness (the number of different species) in balanced healthy communities can negatively affect certain situations, such as overgrowth of invasive taxa, habitat fragmentation, and disturbance [45,46,47]. Thus, a drop in species richness in the PB ileum mucosa community, with the increase in diversity, could have community stabilising benefits while reducing the number of species that have access to mucosa and epithelial cells.

PB increased the abundance of *Firmicutes* while decreasing the abundance of *Proteobacteria* in the caecum, as seen in Figure 4. The LEfSe plot in Figure 6 also indicates a lower abundance of *Proteobacteria* in PB-treated ileum. The gut microbiota is normally composed of *Proteobacteria* in low numbers, but an overgrowth could result in inflammatory responses and intestinal imbalances [48]. The significant alteration of *Proteobacteria* in our dataset results from the significant alteration in *Gammaproteobacteria* (Figure 4 and Figure 6). The *Gammaproteobacteria* class is the home of many human and animal pathogenic genera, like *Salmonella*, *Escherichia*, *Pseudomonas*, *Vibrio*, *Yersinia*, *Legionella*, *Klebsiella*, *Haemophilus*, etc., and contains some of the historically worst pathogens like *Vibrio cholerae* (cholera) and *Yersinia pestis* (plague) [49]. The presence of *Salmonella* in poultry farms is considered a food safety risk due to its high pathogenicity in both humans and birds [50]. *Salmonella* infections result in decreased appetites, dehydration, and diarrhoea, subsequently impacting bird performance [51]. *E. coli* is an important pathogen responsible for colibacillosis, which can affect multiple organ systems [52]. *Pseudomonas* can become opportunistic pathogens that cause respiratory, skin, and ear infections in poultry. Severe cases of *Pseudomonas* infections may even result in septicemia [53]. The genera from the *Gammaproteobacteria* class in our dataset included *Escherichia*, *Parasutterella*, *Gallibacterium*, a range of unknown *Enterobacterales*, and other unknown genera. The fact that we are not detecting individual genera from this class (Figure 4) as differentially abundant indicates that a high significance of PB-driven reduction in *Gammaproteobacteria* as a class in both caecum and ileum likely comes from a number of marginal reductions in multiple genera from this class, which may suggest that PB is targeting common functions associated with this class of bacteria. This could be very promising and deserves further detailed investigation.

We also observed (Figure 6 and Figure 8) reduced *Clostridium sensu stricto 1* abundance in the ileum and ileum mucosa, with LefSe analysis associating this genus with the unsupplemented control. *C. perfringens*, the causative agent of necrotic enteritis, belongs to this group of *Clostridia* [54]. Pathogenic *Clostridia* like *C. difficile* and *C. botulinum* cause muscle degeneration, paralysis, and even death [55]. Hence, a reduced abundance of this genus benefits bird health. Another significant observation is the reduced presence of mucin degraders like *Enterococcus* and *Clostridium* in the ileum mucosa of PB-treated birds. The mucin degraders possess enzymes like glycosidases and sialidases that break down glycosidic bonds and cleave sialic acid residues in the mucin structure [56,57]. Mucin is an important component of the gut that serves as a protective barrier that neutralises pathogens, provides a foundation for beneficial microbes, and prevents the entry of harmful substances into underlying tissues [58]. Ileum mucosa reduction in strict clostridia and *Enterococcaceae* coupled with an increase in *Lactobacillus* and *Faecalibacterium* (Figure 8), a genus known to confer significant benefits to intestinal health [59], is also an indicator of a higher level of epithelial protection provided with this extra amount of glycans supplemented as PB as supported by the histomorphology data. The ability of PB to reduce access of *Enterococcaceae* to epithelia should be further investigated. *Enterococcus*-associated diseases are aggressively emerging in poultry, documented as rapidly increasing in France [60], and resulting in major disease outbreaks in Australia [61].

The PB-treated birds showed an increase in the abundance of phylum *Firmicutes* in the caecum. The phylum *Firmicutes* consists of genera *Faecalibacterium*, *Rumminococcaceae*, *Lachnospiraceae*, etc., which also increased in abundance in the ileum and ileum mucosa. *Firmicutes* contribute towards the production of short-chain fatty acids (SCFAs) in the gut [62]. SCFAs, like butyrate, propionate, and acetate, help maintain the intestinal barrier [63], serve as energy sources for colonocytes, and even regulate the immune response in the gut along with several other functions [64]. Knowing that glycans are the main source of SCFA production, it is possible that this effect is direct via providing more nutrients for SCFA production or indirect via beneficial microbiota modulations.

In all sections, PB-treated birds also showed an increased abundance of *Streptococcus*. *Streptococcus* is a diverse genus that includes neutral gut commensal, pathogenic, and probiotic species in poultry; thus, the presence of *Streptococcus* in the gut of healthy chickens does not indicate the presence of pathogenic strains. Probiotic streptococci reportedly influence bird performance by improving feed efficiency and enhancing body weight [65]. A study on probiotic influences in broilers observed the capacity of probiotic *Streptococcus* in reducing pathogenic *Campylobacter jejuni* colonisation via competitive exclusion [66]. *Streptococcus thermophilus* and other probiotic bacteria can also help in immune response [67]. On the other hand, if the flock had issues with *Streptococcus gallolyticus* associated with systemic infections, including septicemia and endocarditis in poultry, supplementation of such flocks with PB should be avoided until more in vitro experiments can confirm if this promotion of *Streptococcus* extends to *S*. *gallolyticus*.

The *Lactobacillus* genus was significantly higher in PB-treated birds in both ileum and ileum mucosa. Probiotic strains like *Lactobacillus acidophilus* and *Lactobacillus plantarum* assist in immune modulation and stabilising gut microbiota [67,68]. In gnotobiotic chicks, it has been observed that *L. acidophilus* can reduce the presence of pathogenic *E. coli* in the gastrointestinal tract [69]. Probiotic *Lactobacillus* strains can also improve nutrient absorption in broilers [70].

Histological analysis of ileal morphology showed significant differences between the two groups. Although villi were higher in the control group, treated group villi had larger villi surface area, which implies a greater surface for absorption and thus better feed utilisation. Deeper crypts present in the ileum of PB treated group imply a more rapid tissue regeneration process, allowing villi renewal in cases of inflammation caused by either pathogens or their toxins [71]. Histological analysis of liver tissue showed normal tissue structure and the absence of pathological changes. The only significant liver difference between the two groups was the significantly larger area of congested sinusoids in the control group. Previous studies of probiotic and prebiotic supplementation showed that probiotics can alter hepatic sinusoid congestion in poultry [72,73].

The results from this histological analysis suggest that the treatment positively affected liver health by reducing the area of congested sinusoids compared to the control group. This could indicate that the treatment successfully alleviated the congestion, promoted better blood flow, and likely improved the liver tissue’s overall health [74]. Improved blood flow in the liver can have a range of benefits, including the liver receiving more oxygen and nutrients, which can enhance its metabolic activities and especially the detoxification and processing of waste, including microbial toxins [75]. Better blood flow can stimulate liver cell regeneration and repair processes, crucial for recovering from liver injuries or toxin damage. Additionally, liver plays a significant role in immune function [75]. Improved blood flow can support the liver’s immune-related functions, such as removing pathogens and foreign substances.

Based on the histologically confirmed significant improvements in the ileum and liver morphology that would overall result in better nutrient absorption, ileum regeneration and repair and histomorphological improved liver health, these improvements likely helped delay the onset and reduce severity and mortality during infectious bronchitis outbreak in the treated birds. A healthier liver could improve immune function, as the liver plays a significant role in the body’s immune response [75]. A well-functioning liver may better detect and clear pathogens from the bloodstream, including the infectious bronchitis virus, leading to a more effective immune response. Improved liver health helps reduce systemic inflammation, which can be beneficial in combating infectious diseases. A healthy liver contributes to overall body health and vitality [75]. Deeper crypts and larger villi surface area in the ileum would contribute to superior nutrient absorption in the PB-treated birds, better regenerative capacity, and maintenance of the intestinal lining, which indicates enhanced gut barrier function and reduced leaky gut, reduced intestinal inflammation, and enhanced intestinal immune function. A healthier gut environment, characterised by larger villi and deeper crypts, may lead to a more robust gut-associated lymphoid tissue (GALT), improving the gut’s ability to mount an immune response against pathogens like the IB virus.

This study, conducted in a commercial setup, provides a realistic view of birds under high production stress conditions unlikely to be replicated in research facilities due to stricter animal welfare standards. However, controlling all variables and collecting accurate performance data in a commercial setup can be challenging. While controlled research setups can provide statistically more relevant data, they may not fully reflect the conditions experienced by birds in the industry. Our study demonstrates this by showing the performance of birds and PB under extreme conditions, including limited pathogen control, lower biosecurity, and higher disease outbreak incidence. Therefore, a combination of commercial and research facility trials is necessary to comprehensively explore the effects of precision biotics on broiler gut microbiota.

## 5. Conclusions

The manipulation of gut microbiota for enhancing chicken productivity, welfare, and health is emerging as a popular area of poultry research. This study extends beyond the current knowledge of alternative interventions like probiotics, prebiotics, and phytogens, focusing on the targeted modulation of microbial metabolic pathways using precision glycans. This study explored the efficacy of a precision glycan in modulating the gut microbiota, a critical factor in chicken welfare, health, and weight gain. The improved average daily weight gain and feed conversion ratio highlighted the efficacy of the supplement in promoting growth performance under high-stress commercial conditions. The significant delay in the onset of infectious bronchitis, with a substantial reduction in mortality rates in precision glycan-treated birds, underscored the potential of this intervention in enhancing disease resistance. Notably, the alteration in microbial communities, characterised by an increase in the phylum *Firmicutes* and a decrease in the phylum *Proteobacteria*, indicated a healthier gut microbiome balance. Concurrently, there was a notable decrease in potential pathogenic genera, including *Gallibacterium*, *Escherichia*, and *Clostridium*. Larger villi surface area and deeper crypts in the ileum suggested enhanced nutrient absorption and tissue regeneration capabilities.

## Figures and Tables

**Table 1:** Performance data for day 0 to day 38.

Performance	Control	PB.A	PB.B	SEM	*p*-Value
Body weight (g)	2578.86 ^b^	2662.18 ^a^	2658.25 ^a^	10.43	0.0003
Weight gain (g)	2533.61 ^b^	2617.58 ^a^	2612.19 ^a^	10.42	0.0003
Average daily gain (g/day)	67.86 ^b^	70.06 ^a^	69.95 ^a^	10.44	0.0003
Feed intake (g)	4353.68 ^a^	4226.04 ^b^	4165.34 ^b^	7.31	0.0023
Average daily feed intake (g/day)	114.57 ^a^	111.21 ^b^	109.61 ^b^	7.32	0.0023
FCR	1.69 ^a^	1.59 ^b^	1.57 ^b^	12.69	<0.0001
% Liveability 0–38 days	69.80 ^b^	87.05 ^a^	87.46 ^a^	7.76	0.0017
% Mortality 0–38 days	30.20 ^a^	12.95 ^b^	12.55 ^b^	7.76	0.0017
%CV BW	7.78	7.37	6.84		
EPEF	240.48	383.94	390.27		

^a, b^ = means with different superscripts on the same row differ significantly (*p* < 0.05). EPEF = European Production Efficiency Factor.

**Table 2:** PERMANOVA significance using weighted and unweighted UniFrac distance.

Source	Distance	*p*-Value	Significance
Origin (caecum, ileum, ilum mucosa)	UWU	<0.001	***
Treatment (CT vs. PB)	UWU	<0.001	***
Shed (CT, PB1, PB2)	UWU	0.007	**
Origin	WU	<0.001	***
Treatment	WU	0.06	ns
Shed	WU	0.025	*

ns = not significant; WU = weighted UniFrac; UWU = unweighed UniFrac. * = *p* < 0.05, ** = *p* < 0.01, *** = *p* < 0.001.

## Data Availability

The raw sequence data is available from NCBI SRA database with accession number PRJNA1054663 (https://www.ncbi.nlm.nih.gov/bioproject/1054663, accessed on 12 December 2023).

## References

[1] Korver D.R. (2023). Review: Current challenges in poultry nutrition, health, and welfare. Animal.

[2] Bohrer B.M. (2017). Review: Nutrient density and nutritional value of meat products and non-meat foods high in protein. Trends Food Sci. Technol..

[3] Shahbandeh M. Global Chicken Meat Production 2022 & 2023, by Selected Country. https://www.statista.com/statistics/237597/leading-10-countries-worldwide-in-poultry-meat-production-in-2007/.

[4] AgriFutures Australia AgriFutures Chicken Meat. https://agrifutures.com.au/rural-industries/chicken-meat/.

[5] Conraths F.J., Werner O., Methner U., Geue L., Schulze F., Hanel I., Sachse K., Hotzel H., Schubert E., Melzer F. (2005). Conventional and alternative housing systems for poultry—Point of view of infectious disease medicine. Berl. Munch. Tierarztl. Wochenschr..

[6] Ricke S.C., Rothrock M.J. (2020). Gastrointestinal microbiomes of broilers and layer hens in alternative production systems. Poult. Sci..

[7] Van Boeckel T.P., Brower C., Gilbert M., Grenfell B.T., Levin S.A., Robinson T.P., Teillant A., Laxminarayan R. (2015). Global trends in antimicrobial use in food animals. Proc. Natl. Acad. Sci. USA.

[8] Al Sattar A., Chisty N.N., Irin N., Uddin M.H., Hasib F.M.Y., Hoque M.A. (2023). Knowledge and practice of antimicrobial usage and resistance among poultry farmers: A systematic review, meta-analysis, and meta-regression. Vet. Res. Commun..

[9] Economou V., Gousia P. (2015). Agriculture and food animals as a source of antimicrobial-resistant bacteria. Infect. Drug Resist..

[10] Deehan E.C., Yang C., Perez-Munoz M.E., Nguyen N.K., Cheng C.C., Triador L., Zhang Z., Bakal J.A., Walter J. (2020). Precision microbiome modulation with discrete dietary fiber structures directs short-chain fatty acid production. Cell Host Microbe.

[11] Koropatkin N.M., Cameron E.A., Martens E.C. (2012). How glycan metabolism shapes the human gut microbiota. Nat. Rev. Microbiol..

[12] Blokker B., Bortoluzzi C., Iaconis C., Perez-Calvo E., Walsh M.C., Schyns G., Tamburini I., Geremia J.M. (2022). Evaluation of a novel precision biotic on enterohepatic health markers and growth performance of broiler chickens under enteric challenge. Animals.

[13] Jacquier V., Walsh M.C., Schyns G., Claypool J., Blokker B., Bortoluzzi C., Geremia J. (2022). Evaluation of a precision biotic on the growth performance, welfare indicators, ammonia output, and litter quality of broiler chickens. Animals.

[14] Davey R. What Are Glycans?. https://www.news-medical.net/life-sciences/What-are-Glycans.aspx#:~:text=Examples%20of%20functionally%20important%20glycans,and%20manners%20within%20the%20cell.

[15] Gagneux P., Hennet T., Varki A., Varki A., Cummings R.D., Esko J.D., Stanley P., Hart G.W., Aebi M., Mohnen D., Kinoshita T., Packer N.H., Prestegard J.H. (2022). Biological functions of glycans. Essentials of Glycobiology.

[16] Miyachi A., Dohi H., Neri P., Mori H., Uzawa H., Seto Y., Nishida Y. (2009). Multivalent galacto-trehaloses: Design, synthesis, and biological evaluation under the concept of carbohydrate modules. Biomacromolecules.

[17] Ponader D., Maffre P., Aretz J., Pussak D., Ninnemann N.M., Schmidt S., Seeberger P.H., Rademacher C., Nienhaus G.U., Hartmann L. (2014). Carbohydrate-lectin recognition of sequence-defined heteromultivalent glycooligomers. J. Am. Chem. Soc..

[18] Behren S., Westerlind U. (2023). Novel approaches to design glycan-based antibacterial inhibitors. Eur. J. Org. Chem..

[19] Cummings R.D., Esko J.D., Varki A., Cummings R.D., Esko J.D., Freeze H.H., Stanley P., Bertozzi C.R., Hart G.W., Etzler M.E. (2009). Principles of glycan recognition. Essentials of Glycobiology.

[20] Pinho S.S., Reis C.A. (2015). Glycosylation in cancer: Mechanisms and clinical implications. Nat. Rev. Cancer.

[21] Kremsreiter S.M., Kroell A.H., Weinberger K., Boehm H. (2021). Glycan-lectin interactions in cancer and viral infections and how to disrupt them. Int. J. Mol. Sci..

[22] Dube D.H., Bertozzi C.R. (2005). Glycans in cancer and inflammation—Potential for therapeutics and diagnostics. Nat. Rev. Drug Discov..

[23] Slovin S.F., Ragupathi G., Adluri S., Ungers G., Terry K., Kim S., Spassova M., Bornmann W.G., Fazzari M., Dantis L. (1999). Carbohydrate vaccines in cancer: Immunogenicity of a fully synthetic globo H hexasaccharide conjugate in man. Proc. Natl. Acad. Sci. USA.

[24] Gilewski T., Ragupathi G., Bhuta S., Williams L.J., Musselli C., Zhang X.F., Bornmann W.G., Spassova M., Bencsath K.P., Panageas K.S. (2001). Immunization of metastatic breast cancer patients with a fully synthetic globo H conjugate: A phase I trial. Proc. Natl. Acad. Sci. USA.

[25] Ragupathi G., Coltart D.M., Williams L.J., Koide F., Kagan E., Allen J., Harris C., Glunz P.W., Livingston P.O., Danishefsky S.J. (2002). On the power of chemical synthesis: Immunological evaluation of models for multiantigenic carbohydrate-based cancer vaccines. Proc. Natl. Acad. Sci. USA.

[26] Krug L.M., Ragupathi G., Ng K.K., Hood C., Jennings H.J., Guo Z., Kris M.G., Miller V., Pizzo B., Tyson L. (2004). Vaccination of small cell lung cancer patients with polysialic acid or N-propionylated polysialic acid conjugated to keyhole limpet hemocyanin. Clin. Cancer Res..

[27] Guzman-Aranguez A., Argueso P. (2010). Structure and biological roles of mucin-type O-glycans at the ocular surface. Ocul. Surf..

[28] Johansson M.E., Phillipson M., Petersson J., Velcich A., Holm L., Hansson G.C. (2008). The inner of the two Muc2 mucin-dependent mucus layers in colon is devoid of bacteria. Proc. Natl. Acad. Sci. USA.

[29] Johansson M.E., Larsson J.M., Hansson G.C. (2011). The two mucus layers of colon are organized by the MUC2 mucin, whereas the outer layer is a legislator of host-microbial interactions. Proc. Natl. Acad. Sci. USA.

[30] Tytgat K.M., van der Wal J.W., Einerhand A.W., Buller H.A., Dekker J. (1996). Quantitative analysis of MUC2 synthesis in ulcerative colitis. Biochem. Biophys. Res. Commun..

[31] Velcich A., Yang W., Heyer J., Fragale A., Nicholas C., Viani S., Kucherlapati R., Lipkin M., Yang K., Augenlicht L. (2002). Colorectal cancer in mice genetically deficient in the mucin Muc2. Science.

[32] Swidsinski A., Loening-Baucke V., Theissig F., Engelhardt H., Bengmark S., Koch S., Lochs H., Dorffel Y. (2007). Comparative study of the intestinal mucus barrier in normal and inflamed colon. Gut.

[33] Walsh M.C., Jacquier V., Schyns G., Claypool J., Tamburini I., Blokker B., Geremia J.M. (2021). A novel microbiome metabolic modulator improves the growth performance of broiler chickens in multiple trials and modulates targeted energy and amino acid metabolic pathways in the cecal metagenome. Poult. Sci..

[34] Fadrosh D.W., Ma B., Gajer P., Sengamalay N., Ott S., Brotman R.M., Ravel J. (2014). An improved dual-indexing approach for multiplexed 16S rRNA gene sequencing on the Illumina MiSeq platform. Microbiome.

[35] Martin M. (2011). Cutadapt removes adapter sequences from high-throughput sequencing reads. EMBnet J..

[36] Bolyen E., Rideout J.R., Dillon M.R., Bokulich N.A., Abnet C.C., Al-Ghalith G.A., Alexander H., Alm E.J., Arumugam M., Asnicar F. (2019). Reproducible, interactive, scalable and extensible microbiome data science using QIIME 2. Nat. Biotechnol..

[37] Callahan B.J., McMurdie P.J., Rosen M.J., Han A.W., Johnson A.J., Holmes S.P. (2016). DADA2: High-resolution sample inference from Illumina amplicon data. Nat. Methods.

[38] Quast C., Pruesse E., Yilmaz P., Gerken J., Schweer T., Yarza P., Peplies J., Glockner F.O. (2013). The SILVA ribosomal RNA gene database project: Improved data processing and web-based tools. Nucleic Acids Res..

[39] Pfeiffer S., Pastar M., Mitter B., Lippert K., Hackl E., Lojan P., Oswald A., Sessitsch A. (2014). Improved group-specific primers based on the full SILVA 16S rRNA gene reference database. Environ. Microbiol..

[40] O’Hara A.M., Shanahan F. (2006). The gut flora as a forgotten organ. EMBO Rep..

[41] Al-Khalaifah H.S. (2018). Benefits of probiotics and/or prebiotics for antibiotic-reduced poultry. Poult. Sci..

[42] Bajagai Y.S., Alsemgeest J., Moore R.J., Van T.T.H., Stanley D. (2020). Phytogenic products, used as alternatives to antibiotic growth promoters, modify the intestinal microbiota derived from a range of production systems: An in vitro model. Appl. Microbiol. Biotechnol..

[43] Dai D., Qiu K., Zhang H.J., Wu S.G., Han Y.M., Wu Y.Y., Qi G.H., Wang J. (2020). Organic acids as alternatives for antibiotic growth promoters alter the intestinal structure and microbiota and improve the growth performance in broilers. Front. Microbiol..

[44] Shehata A.A., Yalcin S., Latorre J.D., Basiouni S., Attia Y.A., Abd El-Wahab A., Visscher C., El-Seedi H.R., Huber C., Hafez H.M. (2022). Probiotics, prebiotics, and phytogenic substances for optimizing gut health in poultry. Microorganisms.

[45] Ma Z.S., Li L., Gotelli N.J. (2019). Diversity-disease relationships and shared species analyses for human microbiome-associated diseases. ISME J..

[46] Bajagai Y.S., Trotter M., Williams T., Costa D., Whitton M., Ren X., Wilson C., Stanley D. (2022). The role of microbiota in animal health and productivity. Anim. Prod. Sci..

[47] Chetcuti J., Kunin W.E., Bullock J.M. (2020). Habitat fragmentation increases overall richness, but not of habitat-dependent species. Front. Ecol. Evol..

[48] Shin N.R., Whon T.W., Bae J.W. (2015). Proteobacteria: Microbial signature of dysbiosis in gut microbiota. Trends Biotechnol..

[49] Williams K.P., Gillespie J.J., Sobral B.W., Nordberg E.K., Snyder E.E., Shallom J.M., Dickerman A.W. (2010). Phylogeny of gammaproteobacteria. J. Bacteriol..

[50] Ehuwa O., Jaiswal A.K., Jaiswal S. (2021). Salmonella, food safety and food handling practices. Foods.

[51] Gast R.K., Porter R.E. (2020). Salmonella infections. Diseases of Poultry.

[52] Panth Y. (2019). Colibacillosis in poultry: A review *J*. Agric. Nat. Resour..

[53] Abd El-Ghany W.A. (2021). Pseudomonas aeruginosa infection of avian origin: Zoonosis and one health implications. Vet. World.

[54] Chaves Hernández A.J. (2014). Poultry and avian diseases. Encycl. Agric. Food Syst..

[55] Boulianne M., Uzal F.A. (2020). Botulism. Diseases of Poultry.

[56] Hoskins L.C., Boulding E.T. (1981). Mucin degradation in human colon ecosystems. Evidence for the existence and role of bacterial subpopulations producing glycosidases as extracellular enzymes. J. Clin. Investig..

[57] Corfield A.P., Wagner S.A., Clamp J.R., Kriaris M.S., Hoskins L.C. (1992). Mucin degradation in the human colon: Production of sialidase, sialate O-acetylesterase, N-acetylneuraminate lyase, arylesterase, and glycosulfatase activities by strains of fecal bacteria. Infect. Immun..

[58] Grondin J.A., Kwon Y.H., Far P.M., Haq S., Khan W.I. (2020). Mucins in intestinal mucosal defense and inflammation: Learning from clinical and experimental studies. Front. Immunol..

[59] Martin R., Rios-Covian D., Huillet E., Auger S., Khazal S., Bermudez-Humaran L.G., Sokol H., Chatel J.M., Langella P. (2023). Faecalibacterium: A bacterial genus with promising human health applications. FEMS Microbiol. Rev..

[60] Souillard R., Laurentie J., Kempf I., Le Caer V., Le Bouquin S., Serror P., Allain V. (2022). Increasing incidence of *Enterococcus*-associated diseases in poultry in France over the past 15 years. Vet. Microbiol..

[61] O’Dea M., Sahibzada S., Jordan D., Laird T., Lee T., Hewson K., Pang S., Abraham R., Coombs G.W., Harris T. (2019). Genomic, antimicrobial resistance, and public health insights into *Enterococcus* spp. from Australian chickens. J. Clin. Microbiol..

[62] Louis P., Flint H.J. (2017). Formation of propionate and butyrate by the human colonic microbiota. Environ. Microbiol..

[63] Liu P., Wang Y., Yang G., Zhang Q., Meng L., Xin Y., Jiang X. (2021). The role of short-chain fatty acids in intestinal barrier function, inflammation, oxidative stress, and colonic carcinogenesis. Pharmacol. Res..

[64] den Besten G., van Eunen K., Groen A.K., Venema K., Reijngoud D.J., Bakker B.M. (2013). The role of short-chain fatty acids in the interplay between diet, gut microbiota, and host energy metabolism. J. Lipid Res..

[65] Owings W.J., Reynolds D.L., Hasiak R.J., Ferket P.R. (1990). Influence of dietary supplementation with *Streptococcus faecium* M-74 on broiler body weight, feed conversion, carcass characteristics, and intestinal microbial colonization. Poult. Sci..

[66] Morishita T.Y., Aye P.P., Harr B.S., Cobb C.W., Clifford J.R. (1997). Evaluation of an avian-specific probiotic to reduce the colonization and shedding of Campylobacter jejuni in broilers. Avian Dis..

[67] Kabir S.M.L., Rahman M.M., Rahman M.B., Rahman M.M., Ahmed S.U. (2004). The dynamics of probiotics on growth performance and immune response in broilers. Int. J. Poult. Sci..

[68] Kabir S.M.L., Rahman M.B., Rahman M.M., Hosain M.Z., Akand M.S.I., Das S.K. (2005). Viability of probiotics in balancing intestinal flora and effecting histological changes of crop and caecal tissues of broilers. Biotechnology.

[69] Watkins B.A., Miller B.F., Neil D.H. (1982). In vivo inhibitory effects of *Lactobacillus acidophilus* against pathogenic *Escherichia coli* in gnotobiotic chicks. Poult. Sci..

[70] Wu X.Z., Wen Z.G., Hua J.L. (2019). Effects of dietary inclusion of *Lactobacillus* and inulin on growth performance, gut microbiota, nutrient utilization, and immune parameters in broilers. Poult. Sci..

[71] Sobolewska A., Bogucka J., Dankowiakowska A., Elminowska-Wenda G., Stadnicka K., Bednarczyk M. (2017). The impact of synbiotic administration through in ovo technology on the microstructure of a broiler chicken small intestine tissue on the 1(st) and 42(nd) day of rearing. J. Anim. Sci. Biotechnol..

[72] Abd El-Ghany W.A., Abdel-Latif M.A., Hosny F., Alatfeehy N.M., Noreldin A.E., Quesnell R.R., Chapman R., Sakai L., Elbestawy A.R. (2022). Comparative efficacy of postbiotic, probiotic, and antibiotic against necrotic enteritis in broiler chickens. Poult. Sci..

[73] Pithva S.P., Ambalam P.S., Ramoliya J.M., Dave J.M., Vyas B.R. (2015). Antigenotoxic and Antimutagenic Activities of Probiotic Lactobacillus rhamnosus Vc against N-Methyl-N′-Nitro-N-Nitrosoguanidine. Nutr. Cancer.

[74] Volin L., Niittyvuopio R., Heiskanen J., Lindstrom V., Nihtinen A., Sahlstedt L., Ruutu T. (2016). Diagnosis of veno-occlusive disease/sinusoidal obstruction syndrome of the liver: Problems of interpretation. Bone Marrow Transplant..

[75] Ozougwu J.C. (2017). Physiology of the liver. Int. J. Res. Pharm. Biosci..

